# Isolation and Identification of *Colletotrichum nymphaeae as a* Causal Agent of Leaf Spot on *Rhododendron hybridum* Ker Gawl and Its Effects on the Ultrastructure of Host Plants

**DOI:** 10.3390/jof11050392

**Published:** 2025-05-19

**Authors:** Yajiao Sun, Yunjing Tian, Jian Liu, Huali Li, Junjia Lu, Mengyao Wang, Shuwen Liu

**Affiliations:** 1 College of Landscape Architecture and Horticulture, Science, Southwest Forestry University, Kunming 650224, China; 18087323192@126.com (Y.S.); 18283331250@163.com (Y.T.); jian927520@163.com (J.L.); 15912938064@163.com (H.L.); 17587020327@163.com (M.W.); 15887642939@163.com (S.L.); 2 Key Laboratory of Forest Disaster Warning and Control of Yunnan Province, Southwest Forestry University, Kunming 650224, China

**Keywords:** *C. nymphaeae*, *R. hybridum* Ker Gawl, leaf spot, ultrastructural observation

## Abstract

*Rhododendron hybridum* Ker Gawl, a widely cultivated horticultural species in China, is highly valued for its ornamental and medicinal properties. However, with the expansion of its cultivation, leaf spot disease has become more prevalent, significantly affecting the ornamental value of *R. hybridum* Ker Gawl. In this study, *R. hybridum* Ker Gawl from the Kunming area was selected as the experimental material. The tissue isolation method was employed in this study to isolate pathogenic strains. The biological characteristics of the pathogens were determined using the mycelial growth rate method. The pathogens’ influence on the host plant’s ultrastructure was investigated using transmission electron microscopy (TEM). *Colletotrichum nymphaeae* was identified as the pathogen implicated in the development of leaf spot disease in *R. hybridum* Ker Gawl across three regions in Kunming City through the integration of morphological traits and phylogenetic analyses of multiple genes (ITS, ACT, GAPDH, HIS3, CHS1, and TUB2). Its mycelial growth is most effective at a temperature of 25 °C. pH and light have relatively minor effects on the growth of mycelium. The preferred carbon and nitrogen sources were identified as mannitol and yeast extract, respectively. Additionally, TEM observations revealed significant damage to the cell structure of *R. hybridum* Ker Gawl leaves infected by the pathogen. The cell walls were dissolved, the number of chloroplasts decreased markedly, starch granules within chloroplasts were largely absent, and the number of osmiophilic granules increased. This is the first report of leaf spot disease in *R. hybridum* Ker Gawl caused by *C. nymphaeae*. The results of this study provide valuable insights for future research on the prevention and control of this disease.

## 1. Introduction

Rhododendron leaf spot is a common foliar disease that primarily causes the withering and defoliation of Rhododendron leaves [1]. In the early stage of Rhododendron leaf spot disease, tiny brown or purplish-brown spots, approximately the size of pinpoints, appear on the foliage. These lesions typically originate at the leaf tips, margins, or near the veins. As the disease progresses to the mid-stage, the spots gradually enlarge and take on circular, elliptical, or irregular shapes. In the late stage, they continue to expand and coalesce into larger patches. In severe cases, the affected leaves may wither and eventually fall off [2]. Although it rarely leads to plant mortality, it significantly diminishes the ornamental value and growth potential of Rhododendron, a widely cultivated garden plant [3]. Yunnan Province, known for its unique geographical and climatic conditions, as well as its abundant Rhododendron resources, has developed a considerable industry around the plant’s cultivation and utilization. However, the frequent occurrence of leaf spot disease severely impacts Rhododendron growth, development, and aesthetic quality, leading to economic losses [4]. Historically, the diagnosis and classification of Rhododendron leaf spot have primarily relied on lesion color (e.g., brown spots, black spots) and symptomatology (e.g., zonate leaf spot) [5]. However, due to the diversity of pathogenic agents, different pathogens can induce similar symptomatology [6]. Additionally, some pathogens exhibit prolonged incubation periods after host invasion, allowing infected seedlings to remain asymptomatic while still carrying the disease, thereby posing a latent risk of transmission [7]. The inability to effectively prevent the spread of infected seedlings in the early stages and the challenges of accurate disease identification later further complicate the effective management of Rhododendron leaf spot. Therefore, precise identification of Rhododendron leaf spot and a deeper understanding of the pathogenic mechanisms underlying its causal agents are essential. These efforts will provide a theoretical foundation for improving diagnostic accuracy and implementing more effective disease control strategies, ultimately reducing the incidence of Rhododendron leaf spot diseases.

The earliest documented case of Rhododendron leaf spot dates back to 2000, when Rivera reported the disease in Argentina, identifying the pathogen as *Pestalotiopsis guepini* [8]. In 2004, Garibaldi employed a combined morphological and molecular biological approach to identify pathogens, revealing that *C. acutatum* could infect Rhododendron in Italy, leading to the occurrence of leaf diseases. This methodology provided more accurate and reliable pathogen identification results [9]. Subsequently, in 2007, Mrazkova identified the pathogen responsible for Rhododendron leaf spot in a Czech park as *Phytophthora citricola* [10]. In the same year, Álvarez discovered that *P. hibernalis* could also infect Rhododendron in Spain [11]. Both researchers uploaded their pathogen sequencing results to NCBI, obtaining accession numbers that served as valuable references for future research. In 2011, Inghelbrecht reported a new species, *Calonectria colhounii*, as a causative agent of Rhododendron leaf spot in Belgium [12]. In 2012, Blomquist observed that *Phytophthora* sp. was causing significant Rhododendron seedling disease in California [13]. During this period, international researchers primarily focused on improving the accuracy and reliability of pathogen identification. Maharachchikumbura proposed a method integrating morphological characteristics with ITS, TUB, and TEF gene sequences for identifying fungi in the genus *Pestalotiopsis* [14]. Both Zhu Wenya and Liu et al. adopted Maharachchikumbura’s approach. Zhu Wenya used this method to identify the pathogen of Rhododendron delavayi leaf spot in Guizhou as *Pestalotiopsis scoparia* [15], while Liu et al. applied it to identify the pathogen of Rhododendron leaf spot in Zhanjiang, Guangdong, as *Pseudocercospora rhododendricola* [16]. A comprehensive review of scholarly research over the years indicates that reports on Rhododendron leaf diseases have emerged both domestically and internationally. Notably, the causative pathogens exhibit significant diversity, primarily belonging to genera such as *Pestalotiopsis*, *Neofusicoccum*, *Botryosphaeria*, *Pseudocercospora*, *Phomopsis*, *Septoria*, *Cercospora*, *Phytophthora*, *Rhytisma*, *Calonectria*, *Alternaria*, and *Colletotrichum*. An in-depth literature review [8,9,10,11,17] reveals that Rhododendron diseases have been documented across a broad geographic range, including Spain, Italy, Belgium, the Czech Republic, Argentina, the United States, and China. Given its susceptibility to multiple diseases, Rhododendron in China has attracted considerable research attention, leading to numerous reports on its pathology. Currently, incidences of Rhododendron leaf spot are not only well-documented but also widely distributed across different regions. Accurate identification of the pathogen species is essential for developing targeted and effective prevention and control strategies, thereby limiting the spread of the disease at its source. This also provides a stronger theoretical foundation for the effective management of Rhododendron leaf spot disease.

Furthermore, following pathogen invasion, significant ultrastructural alterations occur in the host plant’s cells and tissues, which are closely associated with disease initiation and progression. Examining the effects of the pathogen on the ultrastructure of *R. hybridum* Ker Gawl provides valuable insights into its virulence mechanisms and offers a solid theoretical basis for developing more scientifically grounded and effective strategies for disease prevention and control. At present, most researchers’ studies on Rhododendron diseases mainly focus on the isolation and identification of pathogens. However, there are few reports on the virulence mechanisms of the pathogens causing leaf spot disease in *R. hybridum* Ker Gawl. Over an extended evolutionary timescale, pathogens and host plants have coevolved within a prolonged competitive framework, gradually establishing a dynamic equilibrium. The interplay between pathogen virulence and plant resistance is a critical determinant of disease occurrence and severity. The virulence factors of plant pathogens primarily include toxins and cell-wall-degrading enzymes. Toxins, which are toxic secondary metabolites produced by pathogens, play a central role in the initiation and progression of plant diseases. Their modes of action are complex; even at low concentrations, these compounds can disrupt the ultrastructure of host cells, leading to rapid symptom development and, in severe cases, plant death [18]. Among the key pathogenic factors, toxins play a significant role in disrupting the physiological metabolism of host plants. During infection, pathogens engage in a complex series of interactions with host plants, breaching defenses through stomata, lenticels, or mechanical injuries on the plant surface [19]. Upon recognizing the host, pathogens initiate germination, producing germ tubes and various infection structures, such as appressoria, hyphopodia, infection pegs, primary hyphae, and secondary hyphae [20]. Numerous studies document the ultrastructural damage pathogens inflict on host plants, primarily through toxin-induced alterations. The stronger the pathogenicity of the pathogen, the more toxins it will produce [21]. These include extracellular deposit formation, degradation, and disintegration of the cell wall structure. Additionally, plasmolysis caused by plasma membrane damage has been observed, with the membrane showing folding, invagination, and, in some cases, rupture [22]. The inner and outer membranes of chloroplasts gradually disintegrate, the lamellar system swells and distorts, grana disappear, lipid droplet numbers increase, and the overall chloroplast shape transitions from elliptical to spherical. For instance, *Sporisorium reilianum* invades its host by forming appressoria and subsequently spreads both intercellularly and intracellularly to establish colonization [23,24]. According to previous research, infection by maize stalk rot (MSR) leads to significant disturbances in the chloroplasts and subcellular organelle ultrastructure of maize, thereby impairing normal cellular physiological functions. MSR also causes degradation of maize radicle cell walls [25]. Under stress conditions, Hami melons exhibit marked ultrastructural changes, including thinning of the cell wall, chloroplast disintegration, loss of cellular integrity, and the formation of vacuoles [26]. In *Lilium brownii var. viridulum* Baker infected by *Fusarium oxysporum*, the cell walls, plasma membranes, and nuclei of scale cells are severely damaged. Additionally, an accumulation of mitochondria around the nuclei is observed, and infected cells undergo lysis [27]. In sunflowers inoculated with pathogens, TEM reveals hyphal growth in the intercellular spaces and along cell walls. During infection, chloroplasts become deformed, with disordered thylakoid structures, and in some cases, chloroplast collapse is evident [28]. Similar ultrastructural alterations have also been observed in barley following infection by *Blumeria graminis* [29]. Baka’s team [30] investigated the ultrastructural changes in host plants infected by rust fungi, identifying significant alterations in the shape and structure of chloroplasts and nuclei. By utilizing transmission electron microscopy to examine the ultrastructural changes induced by pathogen infection in Rhododendron leaves, we can gain deeper insights into pathogen attachment and invasion mechanisms. This understanding helps clarify the interactions between pathogens and host cells, providing a crucial foundation for developing more effective disease prevention and control strategies, thereby improving the management of Rhododendron leaf spot.

In this study, the pathogens were isolated from the leaves of *R. hybridum* Ker Gawl infected with leaf spot disease. The pathogens were identified through a combination of morphological and molecular biological methods. The study investigates the impact of the pathogen on the ultrastructure of the leaves of *R. hybridum* Ker Gawl, aiming to explore the possible pathogenic mechanisms of the leaf spot disease in *R. hybridum* Ker Gawl.

## 2. Materials and Methods

### 2.1. Disease Investigation of R. hybridum Ker Gawl

Diseased leaves of *R. hybridum* Ker Gawl ‘Yang MeiHong and Ying TaoHong’ exhibiting leaf spot symptoms were collected in *August* 2023 from three locations in Kunming, Yunnan: the World Horticultural Exposition Garden (N 25°08′, E 102°75′), Southwest Forestry University (N 25°05′, E 102°75′), and Cuihu Park (N 25°04′, E 102°71′). The affected plants displayed brown lesions of varying severity on some leaves, with the most severe cases resulting in leaf drop and wilting. Samples were collected from two varieties of *R. hybridum* Ker Gawl at each of the three sites. For each variety, three plants with typical symptoms were selected, and three diseased leaves were collected from each plant. The samples were then stored in an icebox and transported to the laboratory for further analysis. The condition of the naturally infected leaves was examined using an optical microscope, and the tissues exhibiting clear disease symptoms were sectioned for observation. Photographs were taken from the east, west, south, and north directions of the plants for documentation, and the disease severity was graded based on a modified version of the grading standard in [31] (Table 1). The disease incidence and disease index were calculated based on the disease occurrence survey.

The disease incidence is calculated as follows:

Disease incidence = (Number of diseased plants/Number of investigated plants) × 100%

The disease index is calculated using the formula:

Disease index = [∑(Number of diseased plants at each level × Representative value of that disease level)]/(Total number of investigated plants × Representative value of the highest level) × 100%

### 2.2. Pathogen Isolation and Purification

The diseased Rhododendron leaves were first immersed in 75% alcohol for 7 s, followed by 2% sodium hypochlorite for 30 s. The leaves were then rinsed three times with sterile water and air-dried on sterile filter paper. Tissue samples approximately 1.5 cm × 1.5 cm in size were excised from the junction of the diseased and healthy areas and inoculated onto potato dextrose agar (PDA) plates (composed of 20% potato dices, 2% glucose, and 1.5% agar in distilled water) for cultivation. The plates were incubated upside down at 25 °C for 3 d. Once the mycelium showed significant growth, hyphae were picked and transferred to fresh PDA medium. Pure cultures were obtained after 3–5 rounds of purification processes.

### 2.3. Pathogen Morphological Identification

The isolated pathogen was cultivated on PDA medium, and the colony characteristics were observed following the method outlined by Johnson [32]. To observe the pathogen, the small-culture method was employed. Czapek’s medium (2 g of NaNO_3_, 1 g of K_2_HPO_4_, 0.5 g of MgSO_4_, 0.5 g of KCl, 0.01 g of FeSO_4_, 30 g of sucrose, 15–20 g of agar, and 1000 mL of distilled water) was sterilized and poured into Petri dishes, with the plate thickness kept around 1 mm. Small squares measuring 1.5 cm × 1.5 cm were cut using a scalpel and placed on a glass slide. The pathogen mycelium was then picked and placed at the edge of the small square, and a coverslip was applied. The setup was placed in a moist Petri dish to maintain a stable growth environment and incubated at 25 °C with humidity for 3 d. Once the mycelium had grown, it was observed under a microscope, and the morphological characteristics of the conidia were photographed and recorded. The method for conidia induction was based on Wang [33]. The observation results were then compared with those of previously reported pathogens for morphological identification.

### 2.4. Pathogen Virulence Determination

Following Koch’s postulates, the pathogen was cultured for 7 d. Five mycelial plugs, each with a diameter of 5 mm, were taken from the edge of a colony plate exhibiting dense mycelial growth and inoculated into 150 mL of PDB culture medium. The culture was then placed in a shaking incubator at 28 °C and 180 r/min for 7 d. The spore suspension was diluted to a concentration of 1 × 10^6^ CFU/mL using a hemocytometer. Two-year-old healthy *R. hybridum* Ker Gawl ‘Yang MeiHong and Ying TaoHong’ were selected for inoculation. The plants were disinfected by spraying with 75% alcohol for 30 s, then rinsed 3 times with sterile water and air-dried. The leaf surfaces were lightly wounded with a sterile scalpel, and the well-cultured spore suspension was evenly sprayed onto the leaves of the healthy *R. hybridum* Ker Gawl plants. Healthy, wounded Rhododendron plants sprayed with sterile water served as the blank control. Each treatment was repeated 3 times. The plants were placed in a dark, humid greenhouse at 25 °C, and disease progression on the branches was regularly observed and photographed. After the leaves showed disease symptoms, the tissue at the junction of the diseased and healthy areas was reisolated and purified to confirm whether the isolated fungus was the pathogen.

### 2.5. Pathogen Molecular Biological Identification

Genomic DNA of the fungal strain was extracted using a modified CTAB method following the protocol of a commercial fungal DNA extraction kit [34]. PCR amplification was carried out with primers targeting multiple loci, including the internal transcribed spacer (ITS) region [35], actin (ACT) gene [36], glyceraldehyde-3-phosphate dehydrogenase (GAPDH) gene [37], histone (HIS3) gene [38], chitin synthase A (CHS1) gene [36], and β-tubulin (TUB2) gene [39]. Primer sequences used for Rep-PCR amplification are listed in Table 2. For ITS region amplification, the PCR reaction mixture consisted of 12.5 μL of PCR MIX, 9.5 μL of double-distilled water, 1 μL each of ITS1 and ITS4 primers, and 1 μL of template DNA. The thermal cycling conditions were as follows: initial denaturation at 95 °C for 5 min, followed by 35 cycles of denaturation at 94 °C for 30 s, annealing at 52 °C for 45 s, and extension at 72 °C for 50 s, with a final extension at 72 °C for 10 min, and storage at 4 °C. For amplification using other gene-specific primers, the reaction mixture included 15 μL of PCR MIX, 12 μL of double-distilled water, 1 μL each of the forward and reverse primers, and 1.2 μL of template DNA. The thermal cycling conditions were: initial denaturation at 95 °C for 3 min, followed by 34 cycles of denaturation at 94 °C for 40 s, annealing at either 52 °C or 55 °C for 45 s (as determined by the method described by Slippers et al. [40], and extension at 72 °C for 1 min, with a final extension at 72 °C for 10 min and storage at 4 °C. PCR amplification products were submitted to Beijing Qingke Biotechnology Co., Ltd. (Beijing, China) for sequencing. The resulting sequences were analyzed using the NCBI’s GenBank database for BLAST 2.0 comparison (https://www.ncbi.nlm.nih.gov/ (accessed on 1 March 2025), National Center for Biotechnology Information). Reference sequences were selected based on the relevant literature, and a phylogenetic tree was constructed using the maximum likelihood method in MEGA11. Bootstrap analysis was performed with 1000 replicates to validate the phylogenetic relationships and identify the species of the pathogenic fungus.

### 2.6. Research on the Biological Characteristics of the Pathogen

The pathogen *C. nymphaeae*, which affects *R. hybridum* Ker Gawl, was studied by examining its biological characteristics to identify its optimal growth conditions. This research aims to advance our understanding of the pathogen and contribute to the development of effective control strategies.

#### 2.6.1. Effect of Temperature on Mycelial Growth

Seven temperature settings were tested: 5, 10, 15, 20, 25, 30, and 35 °C. Mycelial plugs with a diameter of 5 mm were punched from the edge of a pathogen colony cultured for 7 days and placed on PDA plates for inverted cultivation. Each temperature setting had three replicates. At 5 and 7 days of cultivation, the colony diameter was measured using the cross-method, and the average mycelial growth rate was calculated as follows:

Average mycelial growth rate (mm/d) = (Average colony diameter at 7 d of cultivation − Average colony diameter at 5 d of cultivation)/2.

#### 2.6.2. Effect of pH on Mycelial Growth [41]

Eight pH gradients were tested: 4, 5, 6, 7, 8, 9, 10, and 11. The pH of the PDA medium was adjusted using 1 mol/L NaOH and 1 mol/L HCl. The medium was then incubated at 25 °C. Each treatment had 3 replicates, and the cultivation and data measurement methods remained the same as above.

#### 2.6.3. Effect of Light on Mycelial Growth

Three light conditions were tested: continuous light for 24 h, continuous darkness for 24 h, and a 12 h light/12 h dark alternation. Each treatment had three replicates, and the cultivation and data measurement methods were the same as described previously.

#### 2.6.4. Carbon Source Screening Test

In Czapek–Dox medium without a carbon source, 30 g of glucose, maltose, *D*-xylose, soluble starch, or mannitol were separately added in equal mass to prepare different carbon source media. The carbon-free medium was set as the control. Each treatment had three replicates, and the cultivation and data measurement methods were the same as described above.

#### 2.6.5. Nitrogen Source Screening Test

In Czapek–Dox medium without a carbon source, 2 g of sodium nitrate, peptone, yeast extract, ammonium sulfate, and ammonium chloride were individually added in equal mass to prepare different nitrogen source media. The nitrogen-free medium served as the control. Each treatment was replicated 3 times, and the cultivation and data measurement methods were the same as described above.

### 2.7. TEM Observation of Rhododendron Leaves Infected by Pathogen

Select healthy *R. hybridum* Ker Gawl ‘Ying TaoHong’ leaves and create wounds using an inoculation needle. Apply 30 μL of the DJ9 spore suspension at a concentration of 1 × 10^6^ CFU/mL, also known as toxin (prepared using the same method as described earlier), onto the wounded areas. Place the treated leaves in a moisturized Petri dish and incubate them at 25 °C for 3 to 5 d, then observe the pathological changes. In the control group, distilled water is used instead of the fermentation broth. On the 7th day of treatment, once obvious symptoms appear, collect and prepare the leaf samples for further analysis.

(1) Sample collection: Select mature Rhododendron leaves, ensuring the main vein is avoided. Cut a small leaf piece (1 mm × 1 mm) from the midpoint where the midrib intersects with the leaf. Fix the sample overnight in 2.5% glutaraldehyde for pre-fixation, keep it at room temperature for 2 h, and then store it at 4 °C in a refrigerator.
(2) Fixation: Before observation, rinse the samples three times with 0.1 mol/L phosphate buffer solution (pH 7.4), with each rinse lasting 15 min. Then, fix the samples in a 1% osmium tetroxide solution for 2 h. After fixation, rinse the samples 4 times with 0.1 mol/L phosphate buffer solution, with each rinsing lasting 15 min.
(3) Dehydration: Perform stepwise dehydration using ethanol solutions of increasing concentrations: 50%, 70%, 80%, 90%, 95%, and 100%. Each dehydration step should last for 15 min.
(4) Embedding: Prepare a mixed solution by combining acetone and Epon 618 epoxy resin embedding agent at a 1:1 ratio. Allow the samples to undergo infiltration overnight. Afterward, proceed with polymerization at 60 °C for 48 h. Once polymerization is complete, use an ultramicrotome to cut ultrathin sections with a thickness of 60 to 80 nm.
(5) Staining: Perform double staining using a saturated aqueous solution of 2% uranyl acetate followed by lead citrate. Each staining step should last for 15 min to enhance contrast for electron microscopy observation.
(6) Observation: Examine the prepared ultrathin sections under a transmission electron microscope (JEM-1400Flash) to assess the cellular structure of the Rhododendron leaves. Observe for signs of plasmolysis and analyze ultrastructural changes in organelles such as chloroplasts, mitochondria, and the nucleus. Capture images for further analysis and documentation of structural alterations. The JEM-1400Flash device was produced by JEOL Ltd. in Tokyo, Japan.

## 3. Results

### 3.1. Disease Investigation and Sampling

**Figure 1:**
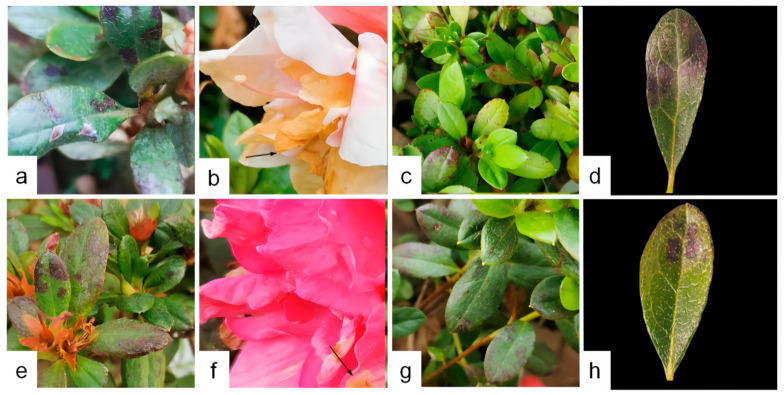
Field Leaf Spot Disease Characteristics of *R. hybridum* Ker Gawl. (**a**–**d**) ‘Yang MeiHong. (**e**–**h**) Ying TaoHong. The observation period was August 2023.

**Figure 2:**
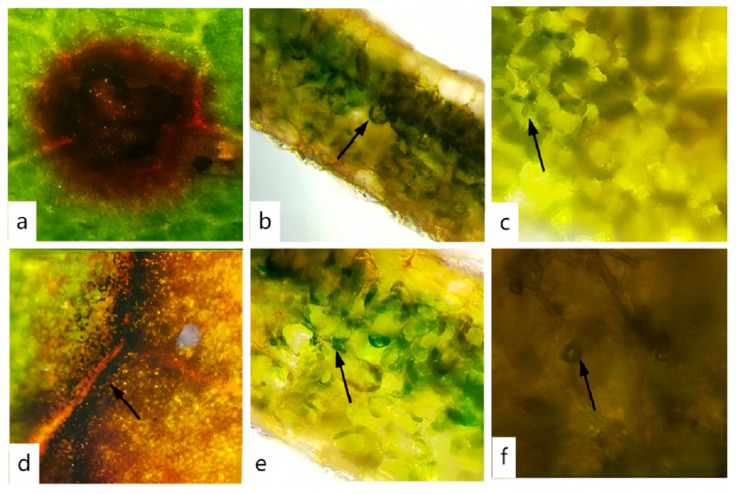
Observation of Diseased Leaf Sections. (**a**,**d**) Observation of leaf spots. (**b**,**c**) The parts indicated by the arrows show that the plant cell structure is damaged and hyphae infect cells through the intercellular layer; (**e**,**f**) The parts indicated by the arrows are suspected to be spores.

A comprehensive disease survey was carried out on Rhododendron hybridum Ker Gawl across three distinct regions in Kunming City. The investigation revealed a widespread occurrence of leaf spot disease, with the overall disease incidence exceeding 60% and reaching up to 100% in severely affected areas. Detailed results of the disease survey are provided in Table 3. During the survey, both varieties, ‘Yang MeiHong and Ying TaoHong’, exhibited brown spots of varying severity, and the symptoms of the lesions were similar (Figure 1). Leaves showing typical and distinct symptoms were collected, placed in an icebox, and transported to the laboratory. The condition of naturally infected leaves was examined under an optical microscope, and tissues with prominent symptoms were sectioned for further observation. The results of these observations are shown in Figure 2. A ring of scorched-like spots was observed at the edges of the lesions. Under a 40× magnification microscope, reddish-brown liquid was detected inside the cells, along with brown and black tissues in the intercellular layer, which were presumed to be mycelial spores (Figure 2e,f).

### 3.2. Screening of Highly Virulent Pathogenic Fungi and Their Morphological Characteristics

**Figure 3:**
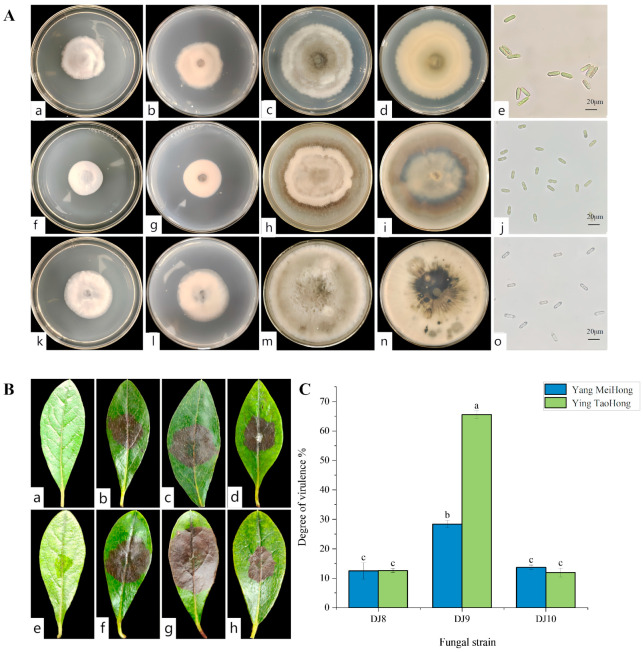
(**A**) Colony morphology and spores of different strains. (**a,b**) The front and back sides of strain DJ8 after growing for 5 days; (**c,d**) The front and back sides of strain DJ8 after growing for 15 days; (**e**) Spore morphology of strain DJ8. (**f,g**) The front and back sides of strain DJ9 after growing for 5 days; (**h,i**) The front and back sides of strain DJ9 after growing for 15 days; (**j**) Spore morphology of strain DJ9. (**k,l**) The front and back sides of strain DJ10 after growing for 5 days; (**m**,**n**) The front and back sides of strain DJ10 after growing for 15 days; (**o**) Spore morphology of strain DJ10. (**B**) The disease symptoms at 7 days after inoculation with the three strains. (**a**) Control group of ‘Yang MeiHong’, (**b**–**d**) respectively show the disease symptoms of DJ8, DJ9, and DJ10 on ‘Yang MeiHong’; (**e**) Control group of ‘Ying TaoHong’, (**f**–**h**) respectively show the disease symptoms of DJ8, DJ9 and DJ10 on ‘Ying TaoHong’. (**C**) The virulence degree of different strains. Different lowercase letters indicate significant differences at the 0.05 level.

Three pathogenic strains, designated DJ8, DJ9, and DJ10, were isolated from symptomatic Rhododendron leaf samples. These strains exhibited distinct colony morphologies when cultured on PDA medium. Preliminary microscopic examination of hyphal and spore characteristics revealed notable differences in spore morphology among the three strains, indicating that they represent different pathogenic species. (Figure 3A). To assess virulence, leaf puncture inoculation assays were conducted using all three strains on healthy leaves of two Rhododendron cultivars, ‘Ying TaoHong’ and ‘Yang MeiHong’. The results show that when the three strains were reinoculated onto the healthy leaves of the two cultivars, brown lesions began to appear approximately 5 to 7 days later, which were consistent with the symptoms of leaf spot disease (Figure 3B). The strain DJ9 induced obvious lesions on the leaves of the “Ying TaoHong” cultivar, with the lesion area accounting for 65.23% of the total leaf surface area. On the leaves of the “Yang MeiHong” cultivar, the strain DJ9 caused a lesion area of 28.43% (Figure 3C). Brown lesions started to appear 4 to 5 days after inoculation, and the lesions were approximately circular. Then, they expanded outward from the wound until they spread across the entire leaf. In contrast, for the strains DJ8 and DJ10, brown spots began to appear 5 to 6 days after inoculation, and the area of the brown lesions was relatively small. This indicates that the strain DJ9 has stronger virulence. Based on these research findings, the strain DJ9 was selected for subsequent analysis.

**Figure 4:**
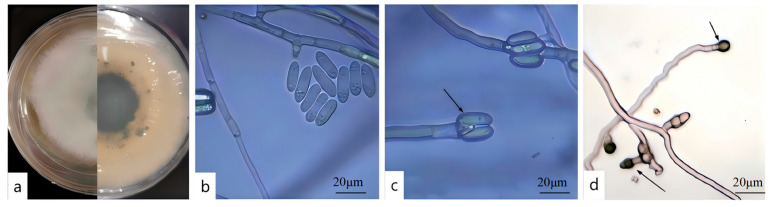
Colony Characteristics and Spore Structure of the Pathogen. (**a**) Front and back of the colony. (**b**) Spore morphology. (**c**) Conidiophores. (**d**) Appendages of spores.

After 7 d of cultivation on PDA, the colony of DJ9 appeared white to light gray, with dense aerial mycelium. Black dots were present on the reverse side, and black circles radiated outward from the center of the colony (Figure 4a). The mycelium cultured on a glass slide for approximately 3 d was examined under a microscope. The conidia were unicellular, colorless, with smooth walls, straight, cylindrical, and had obtuse ends, containing oil globules. The size of the conidia was 14.5–19.2 × 4.2–5.1 μm (average 16.85 × 4.65 μm, n = 40) (Figure 4b,c). The appressoria were single, elliptical, and brown with smooth walls, ovoid to ellipsoidal, and measured 6.4–8.4 × 5.4–6.2 μm (average 7.4 × 5.8 μm, n = 40) (Figure 4d). Based on these observations and in conjunction with references [42,43], strain DJ9 was preliminarily identified as belonging to the genus *Colletotrichum.*

### 3.3. Virulence Determination

**Figure 5:**
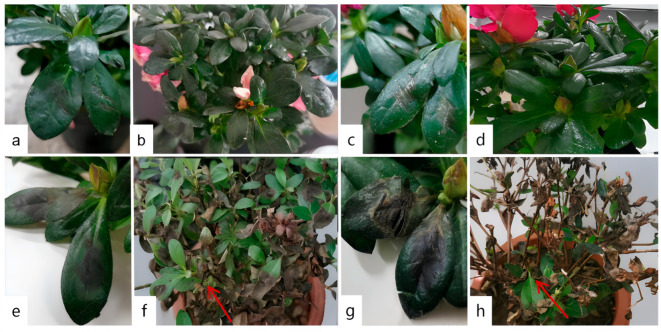
Virulence Observation of *R. hybridum* Ker Gawl Inoculated with the Pathogen. (**a**,**b**,**e**,**f**) ‘Yang MeiHong’. (**c**,**d**,**g**,**h**) ‘Ying TaoHong’. (**a**,**c**) 7 days after inoculation of the control group. (**b**,**d**) 14 days after inoculation of the control group. (**e**,**f**) 7 days after inoculation with strain DJ9. (**g**,**h**) 14 days after inoculation with strain DJ9.

**Figure 6:**
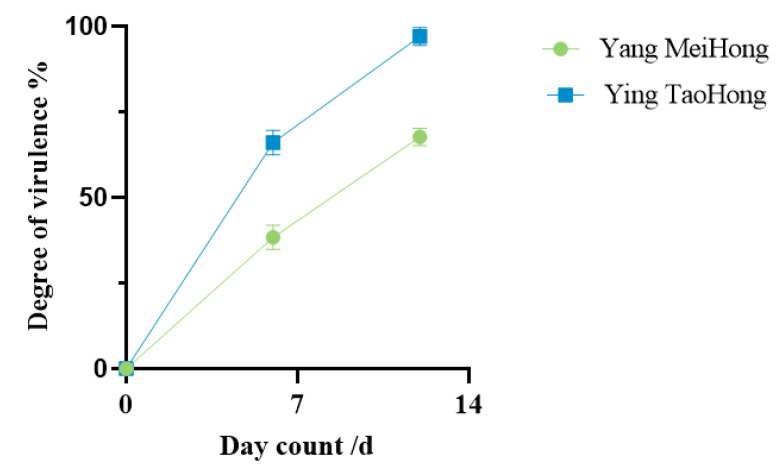
The virulence effect diagrams of *R. hybridum* Ker Gawl at 7 days and 14 days after inoculation with the pathogen.

A spore suspension of DJ9 was evenly sprayed onto the leaves of healthy plants from both varieties. Brown lesions appeared 5–7 d after inoculation, gradually expanding into irregular shapes over time. By 12–14 d post-inoculation, the leaves of the Rhododendron plants showed signs of withering, death, and abscission. The lower and middle leaves withered, and most of the leaves fell off, leaving only the uppermost leaves, while the control plants showed no symptoms. The diseased Rhododendron leaves artificially inoculated showed the same symptoms as those naturally infected in the field, with brown spots appearing on them. (Figure 5). This demonstrated that DJ9 exhibited strong virulence. On the 7th day and 14th day after inoculation with the spore suspension of DJ9, three leaves were randomly surveyed, in three replicates. The percentage of the lesion area to the entire leaf area was measured using ImageJ Fiji software. It was found that the spore suspension of DJ9 exhibited stronger virulence on the *R. hybridum* Ker Gawl ‘Ying TaoHong’ (Figure 6). Diseased leaves were isolated using the tissue isolation method, and the obtained pure strain was compared with the original pure culture strain isolated from the diseased Rhododendron leaves. The strains obtained through two successive rounds of isolation and purification exhibited colony morphology on PDA medium and conidial characteristics consistent with the initial inoculated isolate. According to Koch’s postulates, it can be concluded that DJ9 is the pathogenic agent responsible for leaf spot disease in *R. hybridum* Ker Gawl ‘Yang MeiHong and Ying TaoHong’ in Kunming, Yunnan Province.

### 3.4. Molecular Phylogenetic Tree Analysis of Pathogens

**Figure 7:**
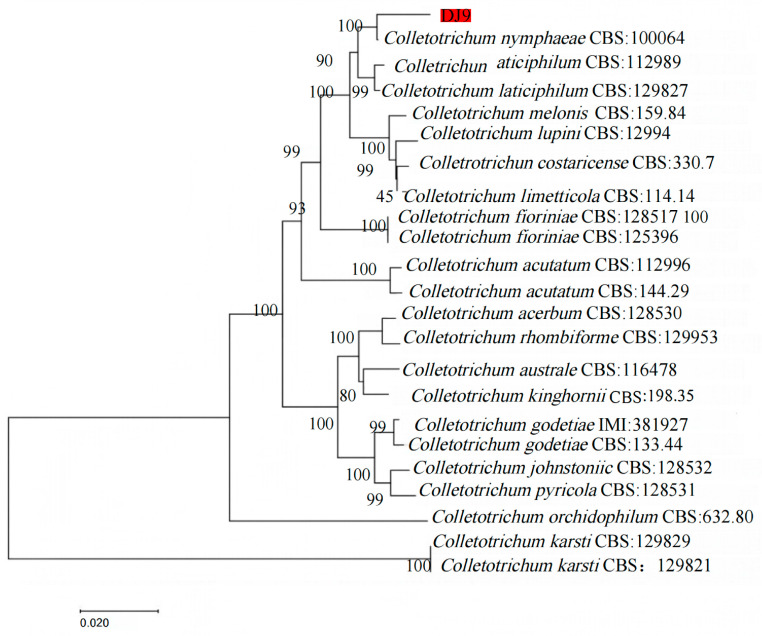
Phylogenetic Tree of Strain DJ9 Based on Multiple Genes ITS, ACT, GAPDH, HIS3, CHS1, and TUB2. The strain marked in red is the target strain DJ9.

Amplification was performed using different genes. The sequencing results were submitted to the NCBI database, and the sequences were analyzed through BLAST alignment. Sequences with over 95% similarity were selected and downloaded. Using MEGA11, a phylogenetic tree for the *R. hybridum* Ker Gawl leaf spot pathogen DJ9 was constructed using MEGA11, based on the concatenated sequences of multiple gene fragments, as shown in Table 4. The analysis revealed that DJ9 clustered with *C. nymphaeae* in the same clade, with a bootstrap value of 100%. Thus, based on both morphological characteristics and phylogenetic analysis, DJ9 was identified as *C. nymphaeae* (Figure 7). The sequences were uploaded to GenBank (https://www.ncbi.nlm.nih.gov/ (accessed on 1 March 2025), National Center for Biotechnology Information), and the accession numbers obtained are OR755663 (ITS), OR767823 (GAPDH), OR767820 (ACT), OR767822 (CHS1), OR767821 (TUB2), and OR767824 (HIS3) (accessed on 1 November 2023, the relevant sequences are shown in the Supplementary Materials). Confirming that the pathogen DJ9 causing leaf spot disease in *R. hybridum* Ker Gawl is *C. nymphaeae* enriches our understanding of the host range of *C. nymphaeae* and provides a foundation for subsequent related research.

### 3.5. Biological Characteristics of the Pathogen

The effects of various environmental conditions, including different carbon and nitrogen sources, temperature, light, and pH, on the growth of the pathogen were examined to investigate the relationship between its occurrence pattern and environmental factors. This study provides a theoretical foundation for the scientific prevention and control of *R. hybridum* Ker Gawl leaf spot disease. The GraphPad Prism 9 statistical software was used to conduct a significant difference analysis of the data from different treatments according to the one-way analysis of variance (ANOVA) model.

#### 3.5.1. Effect of Temperature on the Growth Rate of the Pathogen

**Figure 8:**
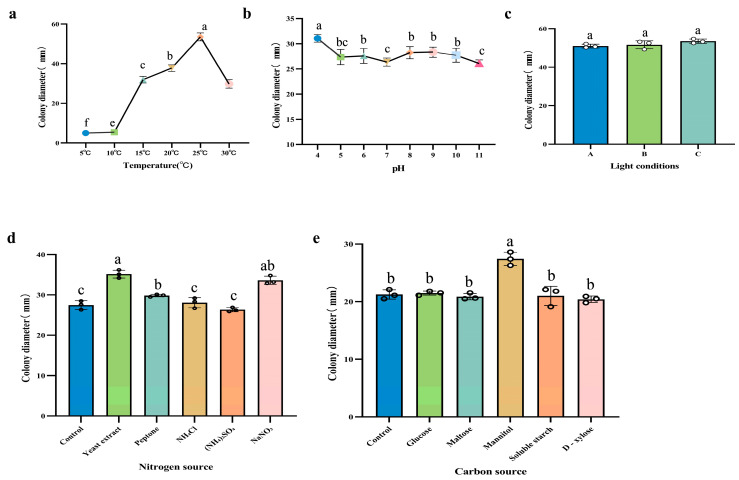
Effects of Different Treatments on the Growth of DJ9 Colonies (**a**) Effects of different temperature treatments on the growth of DJ9 colonies. (**b**) Effects of different pH treatments on the growth of DJ9 colonies. (**c**) Effects of different light conditions on the growth of DJ9 colonies. Among them are A: 24 h continuous illumination, B: 24 h continuous darkness, C: Alternation of 12 h light and 12 h darkness. (**d**) Effects of different carbon sources on the growth of DJ9 colonies. (**e**) Effects of different nitrogen sources on the growth of DJ9 colonies. The vertical lines represent the standard error, and there are significant differences among variables denoted by different letters (*p* < 0.05).

The growth rate of the pathogen under different temperature conditions showed significant variation (Figure 8a). As the temperature increased, the growth rate of strain DJ9 initially increased and then decreased. Strain DJ9 was able to grow within the temperature range of 10–30 °C. At 10 °C, the mycelium showed little growth, and at 15 °C, growth was slow. In contrast, the fastest growth rate was observed at 25 °C, with an average colony diameter of 53.50 mm after 7 d of cultivation, corresponding to a growth rate of 6.60 mm/d.

#### 3.5.2. Effect of pH on the Growth Rate of the Pathogen

After cultivating strain DJ9 on media with pH values ranging from 4 to 11 at 25 °C for 7 d, no significant peak in growth was observed. The growth rate was slightly higher at pH 4, with a colony diameter of 31.08 mm and an average mycelial growth rate of 2.90 mm/d (Figure 8b).

#### 3.5.3. Effect of Light Conditions on the Growth Rate of the Pathogen

Under different light conditions, no significant differences were observed in the growth rate of the pathogen (Figure 8c). For strain DJ 9, the growth rate remained similar under continuous light, continuous darkness, and a 12 h light/12 h dark alternation. The average colony diameter ranged from 51.00 to 53.50 mm, indicating that light conditions had little effect on the colony growth of DJ9.

#### 3.5.4. Effect of Different Carbon Sources on the Growth Rate of the Pathogen

The pathogen was able to grow under different carbon source conditions, with no significant differences observed in the growth rate (Figure 8d). Compared to the control, Czapek’s medium without a carbon source, the growth of strain DJ9 showed no notable differences among the five tested carbon sources. However, when mannitol was used as the carbon source, the growth of strain DJ9 was relatively faster. After 7 d of cultivation, the mycelium grew slowly but densely, with a colony diameter of 27.40 mm and an average mycelial growth rate of approximately 3.90 mm/d.

#### 3.5.5. Effect of Different Nitrogen Sources on the Growth Rate of the Pathogen

Strain DJ9 was able to grow under different nitrogen source conditions (Figure 8e), with no significant differences in the growth rate observed. Among the five tested nitrogen sources, yeast powder and sodium nitrate were the most effectively utilized by strain DJ9. After 7 d of cultivation, the colony diameter was 35.60 mm, and the average mycelial growth rate was 4.20 mm/d. In contrast, the strain showed the poorest utilization of ammonium sulfate, with no significant differences observed in the utilization of the other nitrogen sources.

### 3.6. Ultrastructural Observation of the Host Under Pathogen Stress

**Figure 9:**
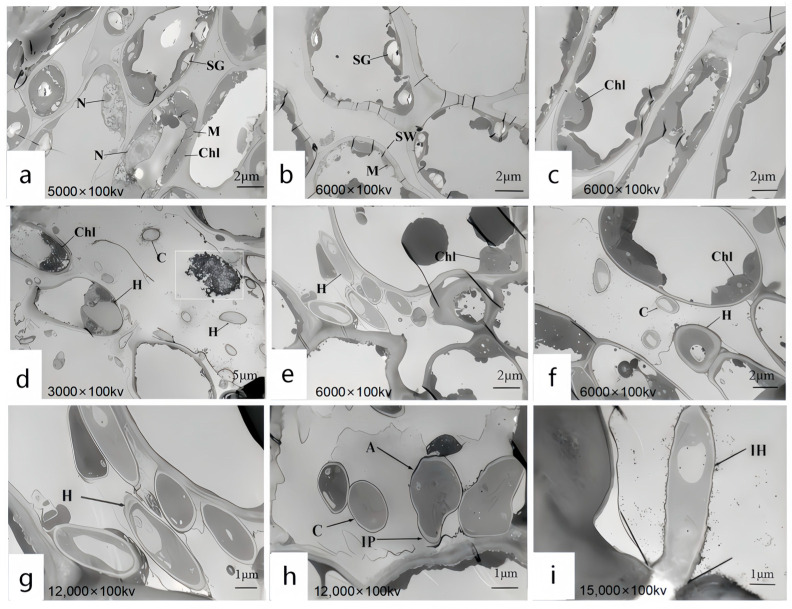
Ultrastructure of *R. hybridum* Ker Gawl Leaf Cells Infected by DJ9 1. (**a**–**c**) The control group consists of *R. hybridum* Ker Gawl leaves treated with sterile water. (**a**) Mitochondria are distributed in the interspaces of chloroplasts. (**b**) There is a relatively large number of mitochondria, and there are numerous starch grains which are relatively large in size. (**c**) Chloroplasts are in contact with the cell wall. (**d**–**f**) The experimental group consists of *R. hybridum* Ker Gawl leaves treated with the spore suspension of DJ9. (**d**) The chloroplasts show a dissolution phenomenon, and the hyphae have successfully invaded the cells. A large number of black osmiophilic granules appear inside the cells. The dissolution phenomenon refers to the destruction of the structural integrity of chloroplasts. The internal granum lamella structure becomes blurred, disordered, and even partially disappears. The outline of the chloroplasts is no longer clear and regular, showing a state of gradual disintegration and dissipation. Some areas exhibit a blank or uneven state. (**e**,**f**) The starch grains and mitochondria disappear, and the chloroplasts show swelling. (**g**) The hyphae and spores adhere to the cell wall. (**h**) It is found that the appressoria have grown into infection pegs, which infect the cell wall, making the cell wall rugged. (**i**) The hyphae infect the cell wall and colonize within the cells. Chl: Chloroplast; SG: Starch grain; CW: Cell wall; M: Mitochondrion; N: Nucleus; OG: Osmophilic granule; Gl: Granum lamella; C: Spore; A: Appressorium; IP: Infection peg; IV: Infection vesicle; H: Hypha.

Upon invasion of plant cells by pathogens, a series of intricate cellular responses are triggered. Using TEM, we observed the distribution of pathogens within the cells and the structural changes in plant cells as a defense mechanism against pathogen invasion. These observations provide direct and crucial insights into the pathogenic mechanisms. The ultrastructure of leaf cells at 7 days post-infection was examined using TEM, and the results revealed significant differences between the experimental group (Figure 9d–i) and the control group (Figure 9a–c) in healthy leaves, particularly in the overall morphological structure of mesophyll cells. Specifically, in Figure 9a–c of the control group, the cell structure of the host plant leaves is intact, with a regular morphology. The cells are generally long and elliptical in shape. Organelles are distributed along the cell wall, and the cytoplasm is evenly distributed. In Figure 8a, mitochondria are distributed in the interspaces of chloroplasts, appearing round or approximately round, and most of the cell nuclei are spherical. In Figure 9b, there are a relatively large number of chloroplasts, which are long and elliptical in shape. Starch grains are distributed within them. There are numerous starch grains, and they are relatively large in size. In Figure 9c, it can be observed that the chloroplasts are in contact with the cell wall with their long-axis planes. They are surrounded by a membrane, and an obvious lamellar structure can be seen. The grana lamellar structure is clear and neatly arranged, and the direction of the lamellae is consistent with the direction of the long axis of the chloroplasts. In Figure 9d–i of the experimental group, the host plant’s cell structure was damaged, and hyphae successfully invaded the cells, leading to a reduction in organelles within the host leaves. In Figure 9e,f, the cell walls and chloroplasts near the infecting hyphae were disintegrated, the starch grains and mitochondria disappeared, and the chloroplasts swelled. In Figure 9d, a large number of black granules were found inside. In Figure 9g, hyphal spores were observed adhering to the cell wall and beginning to degrade the outer membrane of the cell wall. Figure 9h shows the appressorium developing into an infection peg, initiating the infection of the cell wall. In Figure 9i, the hyphae expanded from the intercellular space into the cell, successfully infected the cell wall, and began colonizing within the cell. Scholars have differing opinions regarding the nature of these black granules, suggesting that they may be liposomes, Woronin bodies, cell degradation products, or osmiophilic granules. Previous studies provide relevant descriptions to distinguish these black granules. Mulvey [44] proposed that liposomes, as a membranous delivery system, can significantly improve the bioavailability and solubility of encapsulated substances. Liposomes are spherical vesicles with a bilayer membrane composed of phospholipids, with unilamellar liposomes ranging in particle size from 0.01 to 1 μm. Al-Askar [45] suggested that Woronin bodies are cell structures found near the septa of spores or hyphae, primarily involved in septal function. Mohamed [46] described the diameter of degradation products as approximately 0.5 μm. Based on these descriptions and a comparison with the images, it was determined that the black granules within the chloroplasts were osmiophilic granules. Relevant studies show that after the conidia of *Colletotrichum* land on the surface of the host, infection structures such as appressoria, infection pegs, and hyphae are formed to infect the host, with multiple infection structures often present in the same host [47]. Appressoria are categorized into mycelial appressoria and conidial appressoria, which are brown, thick-walled structures induced under specific conditions. An infection peg can be formed from the appressorium [48]. Appressoria generate extremely high turgor pressure, enabling them to invade the host through an internal–external pressure difference or via the infection peg. Zhang’s team observed during the infection of poplar leaves by *C. gloeosporioides* that the infection peg at the base of the appressorium penetrated the host cuticle and epidermal cell wall, causing the cell wall to rupture and the chloroplasts to disintegrate [49]. The gradual degradation of chloroplasts in this article is consistent with this study.

## 4. Discussion

A disease investigation was carried out on *R. hybridum* Ker Gawl in the World Horticultural Exposition Garden in Kunming, Southwest Forestry University in Kunming, and Cuihu Park in Kunming. The results reveal widespread prevalence of leaf spot disease, with disease incidence exceeding 60% across all areas and reaching up to 100% in regions with severe infection. During the observation, it was noted that the leaf spot disease of *R. hybridum* Ker Gawl primarily affected the leaves. In the early stages, small brown spots appeared on the leaves, which progressively expanded into irregular shapes. As the disease progressed, the lesions darkened to a deep brown and spread throughout the plant. Typical symptomatic samples were collected from the three regions, and tissue isolation and purification were conducted on the infected leaves. Virulence testing identified a strain, DJ9, with relatively high virulence. Comparison of the ITS sequence of strain DJ9 with data from the NCBI database revealed that DJ9 belonged to the genus *Colletotrichum*. Additionally, by integrating morphological characteristics and the results of multi-gene phylogenetic analysis, the pathogen responsible for leaf spot disease in *R. hybridum* Ker Gawl in Kunming, Yunnan Province, was confirmed to be *C. nymphaeae*. This may provide some references for the systematic classification and evolutionary research of pathogens in plant pathology.

Due to the harmful effects of *Colletotrichum* fungi, they have garnered significant attention from experts in fungal taxonomy, plant pathology, and customs quarantine. As one of the most widespread fungal genera, *Colletotrichum* has a broad host range and global distribution, affecting a variety of plants, including garden plants, fruits, and vegetables. Additionally, *Colletotrichum* has the potential to induce diseases in a variety of plant parts. Anthracnose can infect leaves, fruits, stems, etc.; however, leaves are the most susceptible. Van der Aa first described *C. nymphaeae* in 1978 [50]. In his investigation, Van der Aa determined that *Ramularia nymphaeae* and *Gloeosporium nymphaearum* were synonyms for this species. *C. nymphaeae* belongs to the *Colletotrichum acutatum* species complex. A species complex is the term used to describe the collection of numerous pathogen races within the genus *Colletotrichum*, which are difficult to distinguish due to their similar morphologies. At all times, the genetic classification of the genus *Colletotrichum* has been disorganized. Diverse methods are implemented to identify different strains. The gene loci that have been most frequently employed by previous scholars in the identification of *Colletotrichum* fungi are ACT, TUB2, GAPDH, CHS-1, HIS3, etc. [51]. In recent years, multi-gene sequence analysis has become increasingly applied in fungal taxonomy and phylogenetic research, offering more precise and robust support for the identification of fungal species.

*C. acutatum* invades various plant tissues, including leaf petioles, flowers, and fruits, causing tissue necrosis and eventually leading to typical wilt symptoms in affected plants. Currently, there are relatively few reports on *C. nymphaeae*. Known to cause apple rot [52], anthracnose in strawberry, walnut, olive, and tobacco [53,54,55,56], as well as raspberry leaf spot [57], *C. nymphaeae* is reported here for the first time to cause leaf spot disease in *R. hybridum* Ker Gawl leaf spot. *C. nymphaeae* can produce abundant aerial mycelium within a temperature range of 10–30 °C, with an optimal growth temperature of 25 °C. It shows limited growth above 30 °C, which is consistent with other *Colletotrichum* species, such as *C. truncatum* and *C. camelliae*, whose optimal mycelial growth temperature ranges from 25 °C to 30 °C [58,59]. Furthermore, *C. nymphaeae* can grow within a pH range of 4–10, with no significant differences observed, indicating its broad adaptability to both acidic and alkaline conditions. Light conditions, as well as carbon and nitrogen sources, have minimal impact on mycelial growth, suggesting that *C. nymphaeae* exhibits strong adaptability to various growth conditions.

The ultrastructural changes of the leaf cells of *R. hybridum* Ker Gawl inoculated with the spore suspension of the pathogen causing leaf spot disease were observed using TEM. The infected leaves’ cell tissue structure was damaged, the cell wall was dissolved, the number of chloroplasts was significantly reduced, the majority of the starch grains and mitochondria in the chloroplasts disappeared, and the osmiophilic granules increased, as determined by observation and analysis. Macioszek’s team’s research [60] also demonstrated that the pathogen’s infection of the host plant results in the disappearance of starch grains and a decrease in chloroplasts. Starch grains are essential for the growth and development of plants, as they serve as the primary storage form of photosynthetic products in chloroplasts. Starch grains can continuously supply ATP to plants, drive a variety of life activities, and provide the essential carbon source to facilitate the healthy growth of plants [61]. The fungal cell wall is a dynamic structure that protects fungal cells from changes in osmotic pressure and other environmental stresses, while allowing interactions with the environment and other organisms. The dissolution of the cell wall indicates damage to the cellular organization [46]. The cell wall’s dissolution suggests that the cell tissue has been damaged. The host leaf is colonized by numerous conidia of *C. nymphaeae*. The phenomenon of cell wall dissolution may be associated with the secondary metabolites produced by the pathogen, as it occurs after certain conidia contact the cell wall. In this study, infection by pathogenic fungi led to a reduction in the compactness of the plant tissue cell walls, accompanied by deformation of the cell wall structure. These findings are consistent with those of Gao, Wu, and colleagues, who observed similar changes in wheat [62]. This is essentially in accordance with the conclusion reached by the Minkina team. They emphasized that the pathogen’s infection impedes the host plant’s photosynthesis. The main reason for this is that the pathogen damages the chloroplasts, scatters the structure of the chloroplasts, and eventually leads to the disintegration of the chloroplasts [63]. The Baka team has made significant progress in this research field. The chloroplasts and nuclei of the host plant undergo significant changes following the pathogen’s infection, according to the Baka team’s research. The number of chloroplasts is significantly diminished in comparison to the control group [30]. The Van Kan team [64] also noted in the pertinent report that the pathogen spores secrete cell-wall-degrading enzymes and toxins to inflict damage on the host plant when they come into contact with the plant cells. The Mares team [65], in their study of plants infected by rust fungi, reported a significant reduction in the number of chloroplasts. A similar phenomenon was observed in the present study. Some researchers suggest that the decrease in chloroplast numbers in infected plants may be attributed to impaired chloroplast regeneration following infection [66]. Additionally, Mares et al. found that infection of wheat leaves by the stripe–spot pathogen led to alterations in starch metabolism, with starch grains disappearing. In our study, following pathogen infection, starch grains were absent in the infected plants, consistent with the findings of Mares et al. Osmophilic granules, which result from degradation of the chloroplast membrane structure, were also observed in this study. When plant cells are damaged, there is an increase in the number of osmophilic granules within chloroplasts undergoing disintegration. In contrast, chloroplasts with intact membrane structures typically lack or contain very few osmophilic granules. In the present study, a significant accumulation of osmophilic granules was found within the chloroplasts following pathogen infection, indicating severe damage to the chloroplasts. This disruption likely impaired photosynthesis, contributing to the eventual wilting and apoptosis of the entire plant.

*R. hybridum* Ker Gawl is a highly regarded landscape and potted plant in Asia and other regions. Its ornamental and economic value is significantly diminished by the presence of leaf spot disease. This study represents the first documentation of *C. nymphaeaea* as the causal agent of leaf spot disease in *R. hybridum* Ker Gawl. This discovery significantly expands our understanding of the disease spectrum associated with rhododendron cultivation. Precise identification of the pathogen underpinning leaf spot disease in *R. hybridum* Ker Gawl serves as a crucial foundation for the development of targeted disease management strategies. Based on a comprehensive understanding of the pathogen’s biological characteristics, we can rationally select fungicides, thereby optimizing disease control efficacy. Analysis of ultrastructural changes in host cells enables the early detection of pathogen infection, well before the manifestation of visible symptoms. This early warning capacity allows for the timely implementation of disease control measures. Overall, this research provides essential insights that lay the groundwork for the long-term health and sustainable development of *R. hybridum* Ker Gawl cultivation.

Looking ahead, further research is essential. Subsequent investigation should concentrate on the elucidation of the pathogenic mechanism by which *C. nymphaeae* infects *R. hybridum* Ker Gawl, and an in-depth study of the interaction between the pathogen and the plant should be conducted. Through the combined analysis of the transcriptome and metabolome of host plants with different resistance varieties, the differences in the physiological responses of the leaves of resistant and susceptible plant germplasms during the disease occurrence process are compared. Resistance genes are mined and verified, and resistant varieties are screened out, providing a theoretical basis for the breeding of plant disease-resistant varieties. We can begin by focusing on the key pathogenic components of the pathogen. By targeting the essential virulence factors, novel chemical fungicides with low toxicity and high efficacy could be developed. Additionally, by integrating research on the interaction mechanisms between the pathogen and host, elicitor products capable of inducing systemic resistance in plants can be designed. These products would enhance the plant’s innate immunity, enabling it to better resist leaf spot disease and other potential pathogens.

## 5. Conclusions

In this study, we confirmed the taxonomic identity of the pathogenic fungus through both morphological and molecular biological identification, classifying it within the *C. nymphaeae* species complex. TEM revealed significant damage to the host plant’s cellular structure, including the dissolution of the cell wall, a marked reduction in chloroplasts, the disappearance of most starch grains within the chloroplasts, and an increase in osmiophilic granules. This phenomenon significantly perturbs the normal physiological functions of the host plant cells. Eventually, it is manifested as the discernible symptoms of leaf spot disease on *R. hybridum* Ker Gawl, underscoring the intricate and far—reaching impacts of *C. nymphaeae* infection on the host plants. This is the first report identifying *C. nymphaeae* as the causative agent of leaf spot disease in *R. hybridum* Ker Gawl.

## Figures and Tables

**Table 1:** Disease Grading Criteria.

Disease Grade	Standard
0	no visible lesions
1	The area of lesions accounts for less than 5% of the leaf area
3	The area of lesions accounts for 6–10% of the leaf area
5	The area of lesions accounts for 11–20% of the leaf area
7	The area of lesions accounts for 21–40% of the leaf area
9	The area of lesions accounts for more than 40% of the leaf area

**Table 2:** Rep-PCR Primer Sequences.

Gene	Primer	Sequence (5′-3′)	Length of DNA Fragment
ITS	ITS1	TCCGTAGGTGAACCTGCGG	429 bp
	ITS4	TCCTCCGCTTATTGATATGC	
ACT	ACT-512F	ATGTGCAAGGCCGGTTTCGC	252 bp
	ACT-783R	TACGAGTCCTTCTGGCCCAT	
TUB2	Btub2Fd	GTBCACCTYCARACCGGYCARTG	750 bp
	Btub4Rd	CCRGAYTGRCCRAARACRAAGTTGTC	
CHS-1	CHS-79F	TGGGGCAAGGATGCTTGGAAGAAG	300 bp
	CHS-354R	TGGAAGAACCATCTGTGAGAGAGTTG	
GAPDH	GDF1	GCCGTCAACGACCCCTTCATTGA	150 bp
	GDR1	GGGTGGAGTCGTACTTGAGCATGT	
HIS3	CYLH3F	AGGTCCACTGGTGGCAAG	500 bp
	CYLH3R	AGCTGGATGTCCTTGGACTG	

**Table 3:** Disease Survey of Leaf Spot of Rhododendron hybridum Ker Gawl.in Kunming.

Survey Area	Number of Plants at Each Grade						Disease Index	Incidence Rate
	0	1	3	5	7	9		
Kunming World Horticultural Expo Garden	200	120	75	60	35	20	23.31%	60.79%
Southwest Forestry University, Kunming	0	1	5	4	30	20	78.88%	100%
Kunming Cuihu Park	20	20	40	50	10	10	40.74%	86.66%

**Table 4:** Collection Details of Isolates Utilized in Phylogenetic Analysis and Their GenBank Accession Numbers.

Species	Accession	Gene Bank					
		ITS	*GAPDH*	*CHS*-1	*HIS3*	*ACT*	*TUB2*
*C. acerbum*	CBS:128530 *	JQ948459	JQ948790	JQ949120	JQ949450	JQ949780	JQ950110
*C. australe*	CBS 116478	JQ948455	JQ948786	JQ949116	JQ949446	JQ949776	JQ950106
*C. acutatum*	CBS 112996	JQ005776	JQ948677	JQ005797	JQ005818	JQ005839	JQ005860
*C. acutatum*	CBS 144.29	JQ948401	JQ948732	JQ949062	JQ949392	JQ949722	JQ950052
*C. costaricense*	CBS 330.75 *	JQ948180	JQ948510	JQ948841	JQ949171	JQ949501	JQ949831
*C. fiorinae*	CBS128517 *	JQ948292	JQ948622	JQ948953	JQ949283	JQ949613	JQ949943
*C. fiorinae*	CBS125396	JQ948299	JQ948629	JQ948960	JQ949290	JQ949620	JQ949950
*C. godetiae*	IMI 381927	JQ948438	JQ948769	JQ949099	JQ949429	JQ949759	JQ950089
*C. godetiae*	CBS133.44 *	JQ948402	JQ948733	JQ949063	JQ949393	JQ949723	JQ950053
*C. johnstonii*	CBS 128532	JQ948444	JQ948775	JQ949105	JQ949435	JQ949765	JQ950095
*C.karstii*	CBS:129829	JQ005189	JQ005276	JQ005363	JQ005450	JQ005537	JQ005623
*C.karstii*	CBS:130235	JQ005190	JQ005277	JQ005364	JQ005451	JQ005538	JQ005624
*C. kinghornii*	CBS 198.35 *	JQ948454	JQ948785	JQ949115	JQ949445	JQ949775	JQ950105
*C. laticiphilum*	CBS 112989	JQ948289	JQ948619	JQ948950	JQ949280	JQ949610	JQ949940
*C. laticiphilum*	CBS 129827	JQ948290	JQ948620	JQ948951	JQ949281	JQ949611	JQ949941
*C. limetticola*	CBS 114.14 *	JQ948193	JQ948523	JQ948854	JQ949184	JQ949514	JQ949844
*C. lupini*	CBS 129944	JQ948178	JQ948508	JQ948839	JQ949169	JQ949499	JQ949829
*melonis*	CBS 159.84 *	JQ948194	JQ948524	JQ948855	JQ949185	JQ949515	JQ949845
*C. nymphaeae*	CBS 100064	JQ948224	JQ948554	JQ948885	JQ949215	JQ949545	JQ949875
*C.* *orchidophilum*	CBS632.80 *	JQ948151	JQ948481	JQ948812	JQ949142	JQ949472	JQ949802
*C. pyricola*	CBS 128531	JQ948445	JQ948776	JQ949106	JQ949436	JQ949766	JQ950096
*C. rhombiforme*	CBS 129953	JQ948457	JQ948788	JQ949118	JQ949448	JQ949778	JQ950108

(*) indicates that this strain is the type strain of a species in the genus Anthrax, and its characteristics have been used as standard references for species identification.

## Data Availability

The data from this trial can be found in the document. Reagents, microbial materials, and datasets used, created, and analyzed in the study are available upon request to the corresponding author. The genomic sequencing data mentioned in this study are stored in the Sequence Read Archive (SRA) database of the National Center for Biotechnology Information (NCBI) and can be accessed via the NCBI database. The source data are presented in the form of additional source datasets.

## Supplementary Materials

The following supporting information can be downloaded at: https://www.mdpi.com/article/10.3390/jof11050392/s1, The relevant nucleotide sequences of DJ9 in this study, with accession numbers OR755663 (ITS), OR767823 (GAPDH), OR767820 (ACT), OR767822 (CHS1), OR767821 (TUB2), and OR767824 (HIS3), can be found in the Supplementary Materials
