# Supplementary material for: Isolation and Identification of Colletotrichum nymphaeae as a Causal Agent of Leaf Spot on Rhododendron hybridum Ker Gawl and Its Effects on the Ultrastructure of Host Plants
Source: J Fungi (Basel). 2025 May 19;11(5):392. doi: 10.3390/jof11050392 (PMC12112765; doi:10.3390/jof11050392)
Supplement: Supplementary file 1 [file jof-11-00392-s001.zip › jof-3597851-supplementary.pdf]

DJ9 OR755663(ITS) https://www.ncbi.nlm.nih.gov/nuccore/OR755663
TTGCCTTTCGTTTGGGGGTGACTGCGGAGGGATCACTACTGAGGTACCGCTC
TATAACCCTTTGTGAACATACCTAACCGTTGCTTCGGCGGGCAGGGGAAGCC
TCTCGCGGGCCTCCCCTCCCGGCGCCGGCCCCCACCACGGGGACGGGGCGC
CCGCCGGAGGAAACCAAACTCTATTTACACGACGTCTCTTCTGAGTGGCACA
AGCAAATAATTAAAACTTTTAACAACGGATCTCTTGGTTCTGGCATCGATGAA
GAACGCAGCGAAATGCGATAAGTAATGTGAATTGCAGAATTCAGTGAATCAT
CGAATCTTTGAACGCACATTGCGCTCGCCAGCATTCTGGCGAGCATGCCTGTT
CGAGCGTCATTTCAACCCTCAAGCACCGCTTGGTTTTGGGGCCCCACGGCAC
ACGTGGGCCCTTAAAGGTAGTGGCGGACCCTCCCGGAGCCTCCTTTGCGTAG
TAACTAACGTCTCGCACTGGGATTCGGAGGGACTCTTGCCGTAAAACCCCCA
AATTTTTTACAGGTTGACCTCGGATCAGGTAGGAATACCCACTGAACTTAAGC
ATATCAAAAAGGCGGAGGAAAATTTAAAA

DJ9 OR767822(CHS-1) https://www.ncbi.nlm.nih.gov/nuccore/OR767822
GTTTGGGGCAAAGGGATCTTGGAAGAAGATTCTTATCTGTGTCGTCAGCGAC
GGTCGTGGCAAGATCAACCCCAGAACGCGAGCGCTGCTAGCTGGTATGGGT
GTCTACCAGGAGGGTATCGCGAAGCAACAGGTCAACGGCAAGGATGTCACG
GCCCACATTTATGAATACACCTCTCAAGTCGGCATGACGATCAAGAACGACG
TCGTCACACTGGTCCCCAAGCAGCAGCCTGTTCAGATGCTGTTCTGCTTGAA
GGAGAAGAACTCGAAGAACACCAACTCTCTCACAATGGGTTTCT

DJ9 OR767823(GAPDH) https://www.ncbi.nlm.nih.gov/nuccore/OR767823
TTTTGCCGTCAAACCGACCCTTCATTGAGACGCAAGTACGCTGTGAGTATCA
CCCCACTTTACCCCTCCATCATGATATCACGTCTGCCACGATAACACCAGCTTCGTCGAT
ATCCACGGGAAAAGAGTCGGAGCTAGCACTCTCGACTCTTTTGCCCCAAGGTTTCGAT
TGGGCTTGTTGTAACGACACGACGTGACGCGATCATGCAGAAACAGCCGAGACAAAA
CTTGCTGACAGACAATCATCACAGGCCTACATG
CTCAAGACGGACTCCACCCA

DJ9 OR767820(ACT) https://www.ncbi.nlm.nih.gov/nuccore/OR767820
TGTGCAAGGGCCGGTTTCGCCGGTGACGAGCAGCCCAGAGCTGTCTTCCGTA
AGTTCCCCCTCATCCGCAGACCGCAATCTTCTCCGTCAGGGGTATACGATTTC
GGTCACCCATACCCTAGATTGTTGATTCTAACTGTTCCCTAGCCTCCATTGTCG
GTCGCCCCCGTCACCATGGGTAGGTCTATCTCTTGCTGCGGCAATGCCATCTC
CTGCGCCGCGACCTAACACGTGCACAGAATCAGGATTGGTATGGGCCAAAAG
GGACTCGT

DJ9 OR767821(TUB2)https://www.ncbi.nlm.nih.gov/nuccore/OR767821
TATTTGGCCCCCCCTCAGCCGGTCAATGCGTAAGTATCTCCTGATCTCAACCC
AACAAGCCAGAGTGCGGCGCTAACTTCTTTGAACAGGGTAACCAGATTGGT
GCTGCCTTCTGGTGCGTAGCCAACCGCCAGCGACGCGGCGATTCCGACATTT
GACACGATCTCGTACTGACCTTGGTACAGGCAAAACATCTCTGGCGAGCACG
GTCTCGACAGCAATGGCGTGTATGTCACTCGTCCTCCAGTGTGGCTTCCCCGT

GGACCCAGCAGCTAATCATACCATAGGTACAACGGCACTTCCGAGCTCCAGC
TCGAGCGCATGAGCGTCTACTTCAACGAAGTTTGTTATCCTAGTCCCCCAGTG
TGCAGGCAATCCTATTGACGAATGCTGACCTTCTCACCCAACCAGGCCTCCG
GCAACAAGTACGTTCCCCGCGCCGTCCTCGTCGACTTGGAGCCCGGTACCAT
GGACGCCGACCGTGCCGGTCCCTTAGCACAGCCGTTCCGCCCCGACAACTTT
GTTTTGGGCCAAGTCCGGGAGTTCCGGCCCGCTGGGTAACCTCTTCCGCCCG
GACAACTTTGTTTTCGGCCCAATCTGGACGCCTGC

DJ9 OR767824(HIS) https://www.ncbi.nlm.nih.gov/nuccore/OR767824
CCGCATCCCTCATGGTTAGCTGGGAAATTTCTTGGACTGGATGGTGACACGC
ACCGGCGTGCATGGCGCACAGTGTTGGTGTCCTCGAAGAGGGAGACGAGGT
AAGACTCGACGGACTCCTGAAGAGCGCCAATGGCGGAGGACTGGAAGCGA
AGATCGGACTTGAAGTCCTGGGCGATCTCACGAACCTAGGAGGGGAATGTTA
GACGATGCGAAAGGAGAGGCGTTGGGTGGATCTCAACTTACCAGACGCTGG
AAGGGGAGCTTGCGGATCAGAAGCTCAGTGGACTTCTGGTAGCGACGAATC
TCACGAAGAGCGACGGTACCGGGCTTGTAGCGGTGAGGCTTCTTGACACCTC
CGGTGGAGGGGGCGCTCTTGCGGGCGACCTTGGAGGCGAGCCACCGGCGGG
G
